# Interweaving Cultural Narratives, Honoring Diversity: Indigenous and Western Concepts of Dementia

**DOI:** 10.1093/geroni/igaf122.971

**Published:** 2025-12-31

**Authors:** Jordan Lewis

**Affiliations:** Center on Aging, Thompson School of Social Work & Public Health, University of Hawai’i at Manoa, Honolulu, Hawaii, United States

## Abstract

This paper discusses Indigenous concepts of the causes and meaning of dementia, and how these concepts differ from Western concepts. It employs a two-eyed seeing approach, defined as seeing the strengths of the Western knowledge systems through one eye and the strengths of Indigenous value systems through the other eye; both are critical to seeing the whole picture and blending the two systems together. Drawing on the author’s extensive community-engaged participatory research in North American Indigenous communities, the paper explains key features of cultural narratives of Indigenous peoples, which focus on the person and who they were before the onset of dementia symptoms, emphasizing their status as Elders within families and communities, and show how these narratives many also reflect Western narratives focusing on challenges associated with living with dementia. The author provides examples from community-focused collaborations to show how narrative interweaving can support community members through effective, culturally attuned education about dementia.

